# MRI shows thickening and altered diffusion in the median and ulnar nerves in multifocal motor neuropathy

**DOI:** 10.1007/s00330-016-4575-0

**Published:** 2016-09-21

**Authors:** Wieke Haakma, Bas A. Jongbloed, Martijn Froeling, H. Stephan Goedee, Clemens Bos, Alexander Leemans, Leonard H. van den Berg, Jeroen Hendrikse, W. Ludo van der Pol

**Affiliations:** 1 Department of Radiology, University Medical Center Utrecht, Utrecht, The Netherlands; 2 Department of Forensic Medicine and Comparative Medicine Lab, Aarhus University, Aarhus, Denmark; 3 Brain Centre Rudolf Magnus, Department of Neurology and Neurosurgery, University Medical Center Utrecht, Utrecht, The Netherlands; 4 Image Sciences Institute, University Medical Center Utrecht, Utrecht, The Netherlands

**Keywords:** Magnetic resonance imaging, Diffusion tensor imaging, Multifocal motor neuropathy, Amyotrophic lateral sclerosis, Median and ulnar nerve

## Abstract

**Objectives:**

To study disease mechanisms in multifocal motor neuropathy (MMN) with magnetic resonance imaging (MRI) and diffusion tensor imaging (DTI) of the median and ulnar nerves.

**Methods:**

We enrolled ten MMN patients, ten patients with amyotrophic lateral sclerosis (ALS) and ten healthy controls (HCs). Patients underwent MRI (in a prone position) and nerve conduction studies. DTI and fat-suppressed T2-weighted scans of the forearms were performed on a 3.0T MRI scanner. Fibre tractography of the median and ulnar nerves was performed to extract diffusion parameters: fractional anisotropy (FA), mean (MD), axial (AD) and radial (RD) diffusivity. Cross-sectional areas (CSA) were measured on T2-weighted scans.

**Results:**

Forty-five out of 60 arms were included in the analysis. AD was significantly lower in MMN patients (2.20 ± 0.12 × 10^-3^ mm^2^/s) compared to ALS patients (2.31 ± 0.17 × 10^-3^ mm^2^/s; *p* < 0.05) and HCs (2.31± 0.17 × 10^-3^ mm^2^/s; *p* < 0.05). Segmental analysis showed significant restriction of AD, RD and MD (*p* < 0.005) in the proximal third of the nerves. CSA was significantly larger in MMN patients compared to ALS patients and HCs (*p* < 0.01).

**Conclusions:**

Thickening of nerves is compatible with changes in the myelin sheath structure, whereas lowered AD values suggest axonal dysfunction. These findings suggest that myelin and axons are diffusely involved in MMN pathogenesis.

***Key Points*:**

• *Diffusion magnetic resonance imaging provides quantitative information about multifocal motor neuropathy (MMN).*

• *Diffusion tensor imaging allows non-invasive evaluation of the forearm nerves in MMN.*

• *Nerve thickening and lowered diffusion parameters suggests myelin and axonal changes.*

• *This study can help to provide insight into pathological mechanisms of MMN.*

**Electronic supplementary material:**

The online version of this article (doi:10.1007/s00330-016-4575-0) contains supplementary material, which is available to authorized users.

## Introduction

Multifocal motor neuropathy (MMN) is a rare disorder characterized by progressive, asymmetric and predominantly distal limb weakness without any sensory involvement [1]. The diagnosis of MMN is mainly based on the combination of clinical characteristics and specific nerve conduction abnormalities, i.e. conduction block. MMN is a mimic of the early phases of amyotrophic lateral sclerosis (ALS) and progressive muscular atrophy from which it needs to be distinguished, since the prognosis of MMN is much better than that of motor neuron disorders. Although patients with MMN respond to treatment with intravenous or subcutaneous immunoglobulins [2–4], progressive weakness of arms and hands due to accumulating axonal damage causes severe disability in a subgroup of patients [1, 5].

The pathogenic mechanisms that underlie MMN are incompletely understood. The presence of anti-GM1 IgM antibodies in more than half of the patients may suggest that MMN is caused by anti-GM1 antibody-mediated damage at or in the proximity of the nodes of Ranvier. This likely plays a role in the phenomenon of conduction block and in the demyelination or disruption of the compact myelin structure [2, 6]. Demyelination or disruption of the compact myelin structure represents an alternative pathogenic mechanism that causes MMN [6]. There are few pathological studies of affected motor nerves [7, 8], and there are no animal models for MMN. There is a need for new methodology to elucidate MMN pathogenesis and to eventually improve treatment strategies.

Magnetic resonance imaging (MRI) can be used to study the brachial plexus and peripheral nerves [9, 10]. MRI T1- and T2-weighted images can provide anatomical detail and diffusion tensor imaging (DTI) technique information on the microstructural organization of nervous tissue [11–13] and peripheral nerves [14–17]. This unique combination may help to identify relevant disease mechanisms in patients with MMN. In this study we therefore used MRI and DTI to visualize the median and ulnar nerves in the forearm of patients with MMN and ALS and in healthy controls.

## Materials and methods

### Patient characteristics

We enrolled ten patients with MMN, ten patients with ALS and ten healthy controls at the neuromuscular outpatient clinic of the University Medical Centre Utrecht, a tertiary referral centre for neuromuscular disorders. Patients with MMN and ALS fulfilled diagnostic consensus criteria for definite or probable MMN and the El Escorial criteria for ALS, respectively [4, 18]. All patients with ALS had clinical signs of lower motor neuron involvement (i.e. weakness, atrophy and/or fasciculations) in the forearm or hand. Patients and healthy controls were matched for age and gender. All patients with MMN were on immunoglobulin maintenance treatment and all patients with ALS used riluzole. All patients and healthy controls underwent a standardized clinical examination including muscle strength testing of the wrist, thumb and finger flexion, opponens pollicis, abductor pollicis brevis, finger spreading and adductor pollicis, together with sensory testing. Clinical examinations, electromyogram and MRI studies were performed on the same day. The local institutional review board approved this study and we obtained written informed consent from each subject prior to inclusion.

### Nerve conduction study protocol

Nerve conduction studies were performed using a Nicolet VIKING IV electromyogram machine (CareFusion, Tokyo, Japan) after the limbs were warmed in water at 37 °C for 30 min. One of us (SG) was unaware of the clinical diagnosis and performed nerve conduction studies using a shortened version of a previously published protocol [4], consisting of motor nerve stimulation of the median nerve (recording m. abductor pollicis brevis) and ulnar nerve (recording m. abductor digiti V) on both sides up to the axilla. We used the definition of conduction block as described in the diagnostic consensus criteria for MMN [18]. Axonal loss was defined as a decrease of distal compound muscle action potential (CMAP) below to 2 standard deviations of the lower limit of normal, i.e. a CMAP <3.5 mV for the median nerve and a distal CMAP of <2.8 mV for the ulnar nerve.

### MRI protocol and data acquisition

All subjects underwent MRI of both forearms. Scans were acquired on a 3 Tesla MR system (Achieva, Philips Healthcare, Best, The Netherlands) with a 32-channel phased-array surface coil. Patients were positioned in a prone position with one arm placed above the head as described previously [19]. Patients were repositioned when the other arm was scanned. DTI was performed based on diffusion-weighted spin echo single-shot echo planar imaging in the axial plane with the following parameters: TE = 66 ms, TR = 6,340 ms, SENSE factor 2, FOV 240 × 120 mm^2^, matrix size 160 × 80, 60 slices with thickness = 4.0 mm, resulting in a voxel size of 1.5 × 1.5 × 4.0 mm^3^, half scan 0.69, SPIR fat suppression, b-values 0, and 800 s/mm^2^, NSA = 1, and 15 gradient directions. The total acquisition time was 9:32 min. As an anatomical reference, axial fat-suppressed T2-weighted scans were acquired with the following parameters: TE = 90 ms, TR = 7139 ms, SENSE factor 1.5, FOV 120 × 120 mm^2^, matrix size 240 × 234, slices with thickness of 4.0 mm, and spectral attenuated inversion-recovery fat suppression. One stack was used with 60 slices for both the DTI and the T2-weighted scan. Scans with low quality, evaluated by visual inspection, for example due to movement, were excluded from analysis.

### DTI processing

The DTI data were processed using ExploreDTI (www.ExploreDTI.com) [20]. Images were corrected for subject motion, eddy current-induced distortions and susceptibility artefacts [21, 22]. Diffusion tensors were calculated using the REKINDLE method [23] and diffusion parameters were subsequently obtained, which consisted of (1) the fractional anisotropy (FA), (2) the mean diffusivity (MD), (3) the axial diffusivity (AD), and (4) the radial diffusivity (RD) [14]. A standardized deterministic streamline approach was used to reconstruct the fibre tract [24].

### Fibre tractography and diffusion parameters

**Fig. 1:**
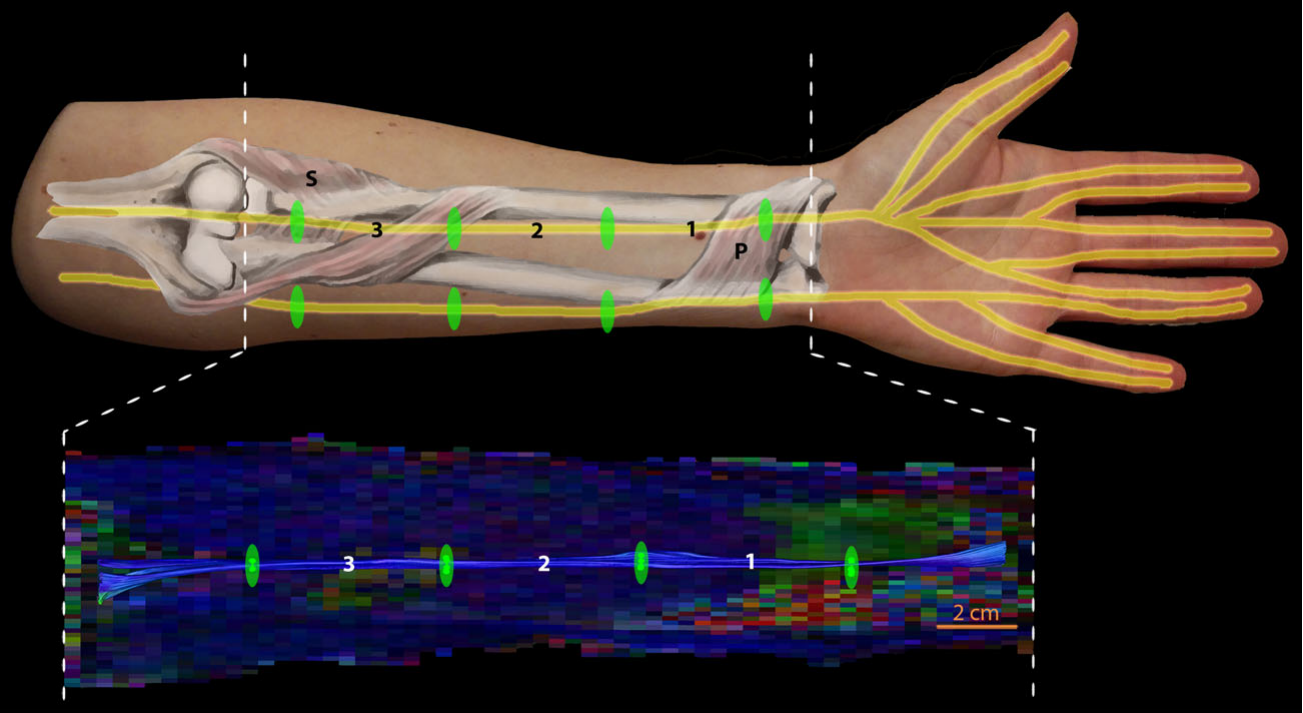
Overview of the region of interest (ROI) positioning along the nerves in the arm (upper image) and the colour-encoded DTI (lower image), where green indicates anterior-posterior, red indicates left-right and blue indicates inferior-superior. The first ROI was placed at the pronator quadratus (P), and the second and third ROIs at one-third and two-thirds of the ulna, respectively, and the fourth ROI was placed at the location of the junction of the supinator (S) with the radius. The tracts were analysed along the entire segment, and segments 1, 2, and 3 individually

To visualize the nerves and extract diffusion parameters, tractography was used. FA range was set to 0.1–0.9, step size of 1 mm, minimum fibre length was set to 100 mm, and the fibre angle was set to 30° per integration step. Fibre tracts, generated by whole volume seeding (1.5 × 1.5 × 1.5 mm^3^), belonging to the forearm nerves were selected by placing ‘AND’ region of interests (ROIs) in four locations in the arm: at the level of the pronator quadratus, one-third of the ulna, two-thirds of the ulna, and the junction of the supinator with the radius [16], as shown in Fig. 1. These locations were chosen as they are relatively easy to locate and therefore provide a reproducible way of selecting the ROIs in each patient in the same way across subjects. ‘AND’ ROIs only select those tracts that run through all the ‘AND’ ROIs. This means that from all the fibre tracts generated from whole volume seeding, only those are selected that span all four predefined locations in the arm. Resulting tracts were used to calculate the diffusion parameters.

To investigate if DTI parameters were biased by the number of tracts and tract length [25], and to investigate to what extent diffusion metrics are distributed homogeneously along the nerve, additional analyses of shorter nerve segments were performed. For this purpose the forearm was subdivided into three segments defined by the ROI positions as described above and shown in Fig. 1. Tracts were selected for each of these individual subsections defined by only two of the four ‘AND’ ROIs and diffusion parameters were calculated for each of the three segments individually.

### Cross-sectional areas

Nerve cross-sectional area (CSA) was assessed on T2-weighted scans, at the predefined four locations in the arm: at the level of the pronator quadratus, one-third of the ulna, two-thirds of the ulna, and the junction of the supinator with the radius as shown in Fig. 1. The mean CSA of each nerve was then calculated based on the average CSA of these four locations.

### Statistical analysis

We used SPSS version 20.0 (SPSS Inc., Chicago, IL, USA) for statistical analysis. A general linear model was used to compare the DTI parameters and the CSA measurements between the three groups. We used Bonferroni correction to correct for multiple testing. The analysis was performed for the whole nerve segment as well as for the individual segments and was based on both median and ulnar nerves. Correction for clustering of the data of scanning two arms in one patient was included into the model, and sex and age were taken into account [13]. Pearson correlation was used to check for correlation between the CSA and the diffusion parameters, and between duration of symptoms and diffusion parameters, where *p* < 0.05 was considered to be significant.

## Results

### Patient characteristics and nerve conduction studies

Patient characteristics are summarized in Table 1. There was a significant difference in duration of symptoms (*p* < 0.001) between patients with MMN and ALS. Distribution and severity of weakness was similar in both patient groups. Nerve conduction studies showed conduction block in seven out of 40 (18 %) nerves, all in patients with MMN. Distal CMAP amplitudes were consistent with axonal loss.

**Table 1:** Characteristics of patients with multifocal motor neuropathy (MMN), amyotrophic lateral sclerosis (ALS) and healthy controls

	MMN (*n* = 10)	ALS (*n* = 10)	Healthy controls (*n* = 10)
Mean age, years (range)	54 (29–67)	53 (40–60)	54 (29–67)
Male (%)	8 (80 %)	8 (80 %)	8 (80 %)
Median duration of symptoms in months (range)	52 (11–124)*	11 (6–34)*	–
Median duration of treatment in months (range)	12 (1–39)	4 (1–24)	–
Weakness lower arm (%)	15/20 (75 %)	15/20 (75 %)	–
Number of conduction blocks (%)	7 (18 %)	0 (0 %)	0 (0 %)
Number of nerves with distal compound muscle action potential < lower limit of normal (%)	6 (15 %)	8 (20 %)	0 (0 %)

*P < 0.001

### MRI protocol and data acquisition

Data quality in patients was lower than in healthy controls. Based on visual inspection, DTI images of 15 out of 60 arms (seven arms of MMN patients, six arms of ALS patients and two arms of healthy controls) had to be excluded due to motion distortion or other MR-related problems resulting in a total of 45 scans of arms that were available for analysis. T2-weighted scans of nine out of 60 arms (three arms of MMN patients, four arms of ALS patients and two arms of healthy controls) had to be excluded, resulting in a total of 51 arms that were used for analysis.

### Fibre tractography

**Fig. 2:**
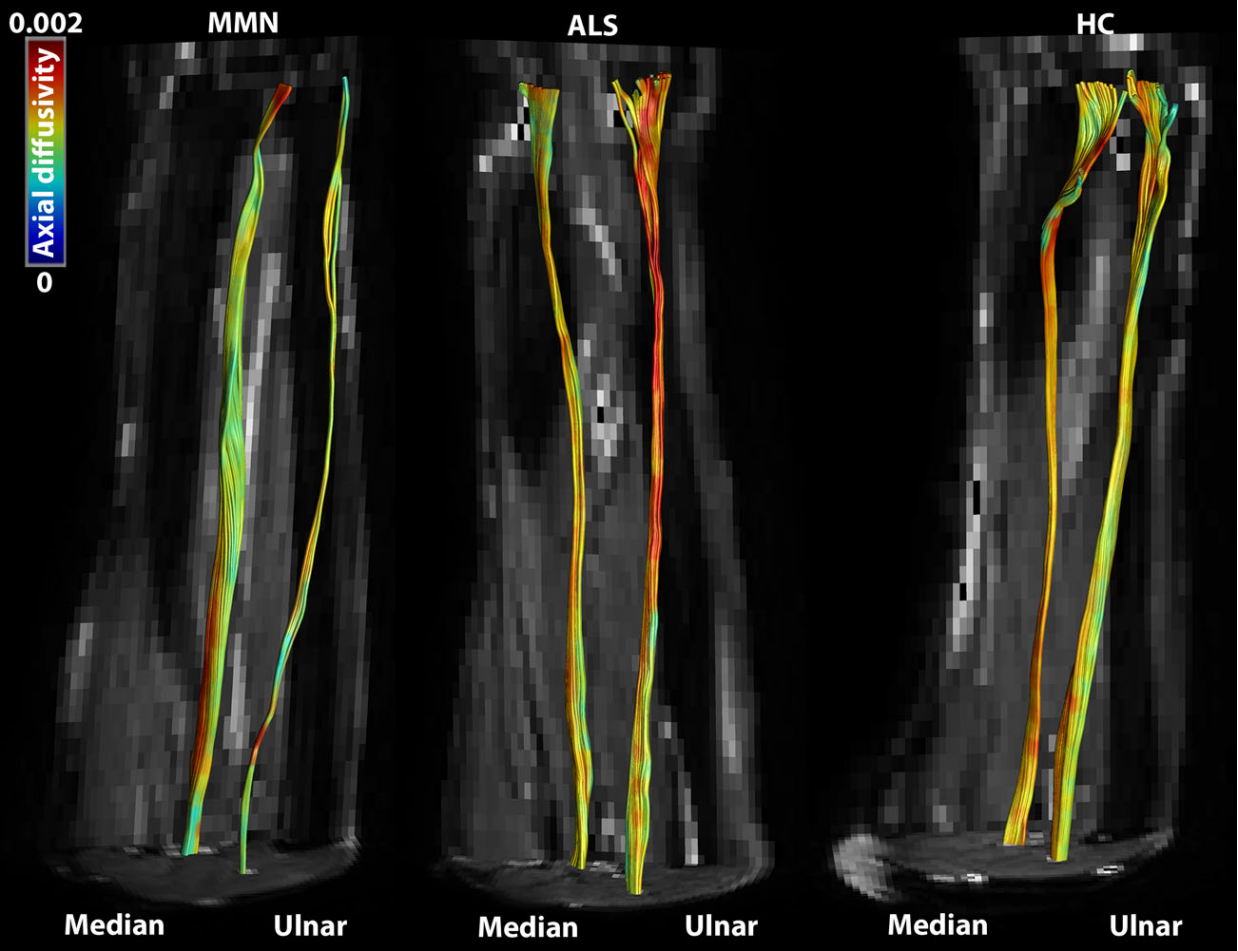
Fibre tractography of the median and ulnar nerve in a multifocal motor neuropathy (MMN) patient, amyotrophic lateral sclerosis (ALS) patient and healthy control (HC). The colour-encoding is according to the axial diffusivity (in units mm^2^/s)

The median and ulnar nerves could be reconstructed with fibre tractography in 40 of the 45 datasets. Figure 2 shows the tracts derived from the median and ulnar nerves in patients with MMN and ALS as well as in a healthy control. In five datasets tracts could not be reconstructed in seven nerves (six nerves of four MMN patients, and one nerve in a healthy control).

In total four nerves with conduction blocks and three nerves with axonal damage remained for analyses in the MMN group, and five nerves with axonal damage in the ALS group.

Supplementary Table 1 shows the number of tracts per nerve and nerve segment. It was not possible to find tracts in three nerves of segments 1 and 3 and in two nerves of segment 2 in patients with MMN, and in one nerve in segment 1 in a healthy control.

### Diffusion parameters

There were no significant differences between the two arms. Therefore, left and right arms were combined in the data analysis. The calculated average diffusion parameters of all tracts belonging to the median and ulnar nerves are summarized in Table 2. There was a significant difference in AD of nerves of patients with MMN (2.20 ± 0.12 × 10^-3^ mm^2^/s) compared to patients with ALS (2.31 ± 0.17 × 10^-3^ mm^2^/s; *p* < 0.05) and to healthy controls (2.31 ± 0.17 × 10^-3^ mm^2^/s; *p* < 0.05). There were no significant differences in FA, MD and RD between the groups.

**Table 2:** Mean diffusion parameters (fractional anisotropy (FA), mean (MD), axial (AD) and radial (RD) diffusivity) with standard deviation (SD) based on both median and ulnar nerves in patients with multifocal motor neuropathy (MMN), amyotrophic lateral sclerosis (ALS) and healthy controls (HC)

	MMN	ALS	HC
FA			
Entire nerve^a^	0.44 ± 0.04	0.43 ± 0.05	0.44 ± 0.04
Segment 1^b^	0.43 ± 0.05	0.43 ± 0.05	0.43 ± 0.04
Segment 2^c^	0.44 ± 0.05	0.43 ± 0.05	0.44 ± 0.05
Segment 3^d^	0.46 ± 0.06	0.44 ± 0.03	0.45 ± 0.04
MD (×10^-3^ mm^2^/s)			
Entire nerve	1.44 ± 0.10	1.52 ± 0.15	1.51 ± 0.14
Segment 1	1.50 ± 0.18	1.46 ± 0.17	1.51 ± 0.16
Segment 2	1.43 ± 0.16	1.49 ± 0.18	1.49 ± 0.14
Segment 3	1.38 ± 0.14^**^	1.50 ± 0.10^**^	1.45 ± 0.11
AD (×10^-3^ mm^2^/s)			
Entire nerve	2.20 ± 0.12^*^	2.31 ± 0.17^*^	2.31 ± 0.17^*^
Segment 1	2.26 ± 0.22	2.22 ± 0.20	2.29 ± 0.20
Segment 2	2.19 ± 0.18	2.27 ± 0.22	2.27 ± 0.15
Segment 3	2.16 ± 0.18^*^	2.30 ± 0.13^*^	2.25 ± 0.14^*^
RD (×10^-3^ mm^2^/s)			
Entire nerve	1.06 ± 0.10	1.13 ± 0.15	1.11 ± 0.14
Segment 1	1.12 ± 0.18	1.08 ± 0.16	1.11 ± 0.15
Segment 2	1.05 ± 0.16	1.11 ± 0.18	1.09 ± 0.14
Segment 3	0.99 ± 0.14^**^	1.11 ± 0.09^**^	1.05 ± 0.11

^a^Whole segment: *n* = 20, *n* = 28 and *n* = 36 for, respectively, MMN, ALS and HC

^b^Segment 1: *n* = 23, *n* = 28 and *n* = 35 for, respectively, MMN, ALS and HC

^c^Segment 2: *n* = 24, *n* = 28 and *n* = 36 for, respectively, MMN, ALS and HC

^d^Segment 3: *n* = 23, *n* = 28 and *n* = 36 for, respectively, MMN, ALS and HC

^*^Significant difference in MMN vs. ALS and controls (p < 0.05)

^**^Significant difference in MMN vs. ALS (p < 0.005)

Results of diffusion parameters of nerves with and without reduced distal CMAP are shown in Table 3. Nerves with reduced distal CMAP (both MMN and ALS patients) showed lower MD and RD values (*p* < 0.05). The AD in these nerves showed a tendency (not significant *p* = 0.083) towards lower values.

**Table 3:** Mean diffusion parameters and cross-sectional areas (CSA) of the median and ulnar nerves with reduced compound muscle action potential (CMAP) amplitudes, i.e. smaller than the lower limit of normal (LLN) reflecting axonal loss, versus nerves with normal CMAP amplitudes

	CMAP < LLN *(N* = 8)	CMAP > LLN *(N* = 76)
FA	0.47 ± 0.05*	0.44 ± 0.04*
MD (×10^-3^ mm^2^/s)	1.40 ± 0.12*	1.51 ± 0.14*
AD (×10^-3^ mm^2^/s)	2.19 ± 0.14	2.30 ± 0.16
RD (×10^-3^ mm^2^/s)	1.00 ± 0.13*	1.12 ± 0.14*
CSA	7.09 ± 1.29	6.48 ± 1.37

* *p* < 0.05

*FA* fractional anisotropy, *MD* mean diffusivity, *AD* axial diffusivity, *RD* radial diffusivity

Segmental analysis of diffusion parameters also showed a significantly lower AD value in segment 3 for MMN patients compared to ALS patients (*p* < 0.005) and healthy controls (*p* < 0.05), and additionally significantly lower MD and RD (*p* < 0.005) values in patients with MMN compared to those with ALS, as shown in Table 2.

### Cross-sectional areas

**Fig. 3:**
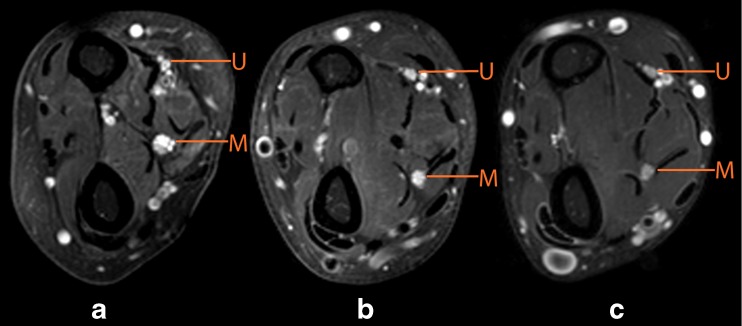
Axial plane of T2-weighted scans of the forearm with the median (M) and ulnar (U) nerves. **a** Multifocal motor neuropathy (MMN) patient with enlargement of the median nerve, (**b**) amyotrophic lateral sclerosis (ALS) patient and (**c**) healthy control

**Fig. 4:**
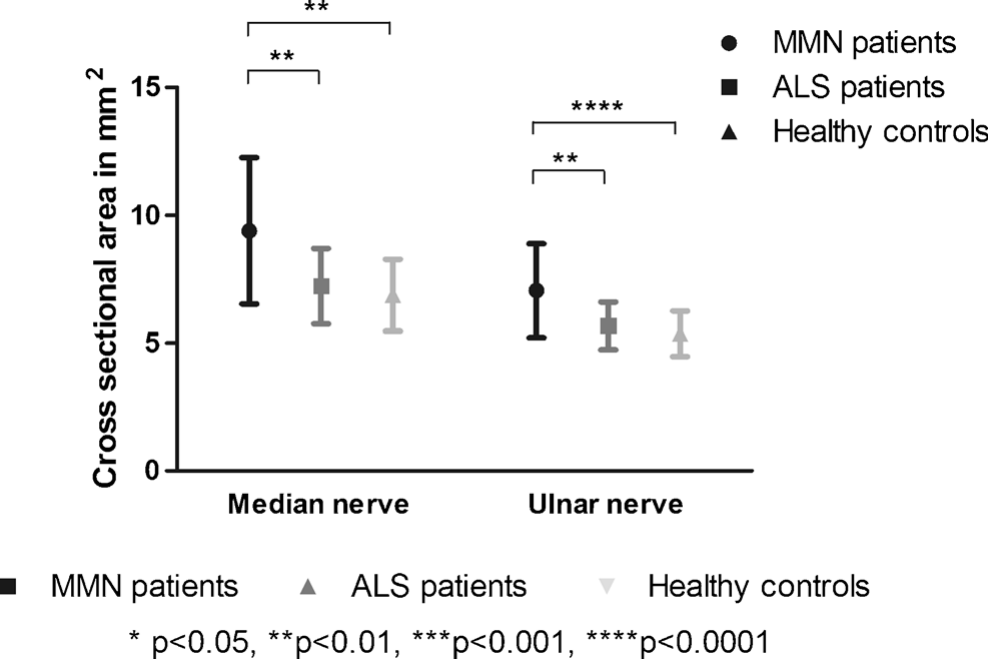
Cross-sectional area of the median and ulnar nerves in the forearm with standard deviation (SD). Multifocal motor neuropathy (MMN) patients differed significantly from amyotrophic lateral sclerosis (ALS) patients and healthy controls

Figure 3 shows representative examples of CSA of the median nerve of a patient with MMN, a patient with ALS and a healthy control on T2-weighted scans. The mean CSA of the median and ulnar nerves on T2-weighted scans were significantly larger in patients with MMN (median = 9.40± 2.87 mm^2^, and ulnar = 7.06 ± 1.84 mm^2^) compared to those with ALS (median = 7.23 ± 1.47 mm^2^, and ulnar = 5.68± 0.93 mm^2^) and to healthy controls (median = 6.88± 1.41 mm^2^, and ulnar = 5.36 ± 0.89 mm^2^) (Fig. 4). There was no correlation between CSA and any of the diffusion parameters (FA, MD, AD and RD) (max *r* = 0.262), or between duration of symptoms and diffusion parameters (max *r* = 0.391). Nerves with reduced CMAP amplitudes did not show a significant difference in CSA compared to nerves with normal CMAP amplitudes (see Table 3). There was no correlation between the CSA and any of the diffusion parameters (max *r* = 0.160) in the nerves with reduced CMAP amplitudes.

## Discussion

This MRI study shows anatomical and diffusion abnormalities in peripheral nerves from patients with MMN. We found significant nerve enlargement in combination with a significant reduction of axial diffusion. These *in vivo* results suggest that pathogenic mechanisms in MMN might affect both the axon and myelin sheath, as was earlier suggested by pathological abnormalities near the site of conduction block [8].

The median and ulnar nerves of MMN patients were 25–30 % larger than those in the healthy controls and ALS patients. The diffuse rather than focal nerve enlargements are in line with high resolution ultrasound studies of peripheral nerves [26, 27] and the brachial plexus [9, 28]. Pathogenic mechanisms underlying MMN therefore seem to affect significant lengths of motor nerves rather than patchy and focal involvement, as suggested by the observed patterns of weakness and conduction block [1, 5].

There are clear indications that nerve thickening on MRI reflects involvement of the myelin sheath. It is a consistent feature of both genetic and acquired demyelinating polyneuropathies, i.e. Charcot-Marie-Tooth type 1 and chronic inflammatory demyelinating polyneuropathy [28, 29]. This is further supported by the occasional pathological observation of onion bulb formation in nerve biopsy studies in MMN [7, 30, 31].

Demyelination is probably not the only pathological mechanism that underlies MMN, since it does not explain all disease characteristics, such as the phenomenon of cold paresis [32]. Findings in the rabbit model for acute motor axonal neuropathy and human motor neuron model for MMN [33, 34] and clinical observations of significant axonal damage in patients with MMN [5, 35] suggest additional pathological mechanisms that directly affect the axon [36]. The DTI findings in this study, in particular the reduced AD values, support this concept. Reduced AD values reflect pathological changes that impair diffusion in the length of the axon and are associated with Wallerian degeneration in animal studies [11, 12, 37]. In a recently developed *in vitro* model of anti-GM1 IgM antibody-mediated damage to human motor nerves, we observed focal widening of the axon that preceded Wallerian degeneration [34]. MRI studies in an ischaemia-model of rat sciatic nerve showed that this process of axonal ‘beading’ was associated with significantly restricted AD and virtually unchanged RD and FA values [38]. The reduced AD values may therefore reflect pathological changes in motor axons of patients with MMN. The subanalysis performed on nerves with reduced CMAP amplitudes shows lower FA, MD and RD values and a tendency toward lower AD in the median and ulnar nerves. This tendency toward lower AD could be associated with a reduction of axon integrity [13]. Reduced MD might be due to disruption of the cytoskeleton, increasing the viscosity [39]. Detailed analysis of the association of conduction block and MRI and DTI abnormalities would be of added value to further explore the pathophysiological mechanisms behind MMN. However, this was not possible due to the low number of conduction blocks in this patient sample, which would make a statistical analysis severely underpowered. This is a topic for future larger scale studies.

Patients appeared more uncomfortable in the prone scanning position, and as a result motion artefacts were more common in patients than in healthy controls. This scanning position was a methodological limitation of this study and resulted in the exclusion of a significant number of scans due to the relatively low quality of this data. During development of the protocol we aimed to obtain a protocol with a sufficiently high resolution to distinguish the nerves and to have sufficient signal-to-noise ratio (SNR), as the SNR, amongst other things, will influence the precision of the DTI metrics [25, 40]. Future development of DTI protocols in the forearm should focus on the right trade-off in SNR, resolution (preferable <1 × 1 mm in plane), and scan time, as SNR and resolution will always come at the cost of increased scan time and thus patient discomfort [25]. Repositioning patients in the supine position and using dedicated arm coils could improve patient comfort and therefore reduce motion artefacts in future studies. This will improve data quality resulting in less data that need to be rejected due to artefacts.

We used a tract-based analysis approach with a minimum length of fibre tracts of 100 mm to exclude aberrant inclusion of muscle fibres. As a consequence, the number of tracts available for final analysis was small, since only a limited number of fibres can be traced over this range. Moreover, the error accumulation over a long tract range can become substantial and may introduce bias [25]. To overcome this problem, we additionally performed segmental analysis, in which smaller segments of the nerves were analysed resulting in more fibre tracts being included. The higher number of tracts allows for better sampling of the data. Segmental analysis showed similar AD changes and additional significant differences in RD values in the proximal segment (segment 3), but not in the distal segments (segments 1 and 2), of the median and ulnar nerves. Decreased RD values further support pathological processes that directly affect the axon, rather than demyelination [11, 41].

An obvious limitation of our study is the number of included patients, which limits its power. Furthermore, the age range of ALS patients did not fully match those of the MMN patients and healthy controls. As ALS and MMN are rare diseases, matching of these two groups is challenging. This was further complicated by the fact that we could only include a selection of ALS patients who were able to lie still in a prone position for a relatively long time. However, mean age, standard deviations and 95 % confidence intervals of ALS patients were similar to those of patients with MMN and healthy controls.

In line with previous studies we found no differences in diffusion parameters between the left and right arms [42]. DTI has not been used extensively to investigate forearm nerves and there are few comparable studies of peripheral nerves of the arm in healthy controls [16] or patients with polyneuropathy [43, 44]. Comparison of previous results with our findings is further complicated by differences in MRI settings (e.g. smaller voxel size and higher b-value), the difference in threshold settings used for tractography (higher FA threshold results in higher FA values [25]) and patient characteristics [43].

Partial volume effects have to be considered when interpreting DTI results in small nerves [45]. Partial volume effects are caused by the voxels located on the edges of the nerves and thus partially contain muscle tissue signal, which has a lower AD, and a higher RD than the nerve. As a consequence, partial volume effects would lower the AD and increase the RD values [19]. However, partial volume effects cannot explain the current findings of lower AD values as nerves of MMN patients had a larger CSA and consequently lower partial volume effects [45]. Partial volume effects could influence the RD values and provide an alternative explanation for the differences found in RD between patients with MMN and controls.

In conclusion, this study shows that MRI and DTI can detect lowered AD and enlarged CSA in patients with MMN compared to ALS and to healthy controls. These results can help to provide insight into pathological mechanisms of MMN. Future studies would be facilitated by improving patient comfort, for example through the use of dedicated arm coils and placing patients in a supine position, which could reduce motion artefacts and thus improve data quality. As a result less data need to be rejected due to artefacts. Comparative DTI studies of patients with MMN and other demyelinating peripheral nerve disorders, such as chronic inflammatory demyelinating polyneuropathy and Charcot-Marie-Tooth type 1A, could help to further clarify the aetiology of MMN.

## Electronic supplementary material

Below is the link to the electronic supplementary material.

Supplementary Table 1 (DOCX 12 kb)
