# Supplementary material for: MRI shows thickening and altered diffusion in the median and ulnar nerves in multifocal motor neuropathy
Source: Eur Radiol. 2016 Sep 21;27(5):2216–24. doi: 10.1007/s00330-016-4575-0 (PMC5374174; doi:10.1007/s00330-016-4575-0)
Supplement: Supplementary file 1 — (DOCX 12 kb) [file 330_2016_4575_MOESM1_ESM.docx]

**Supplementary files**

**Supplementary table 1**: Number of tracts (NT) for the entire tract and segments 1, 2, and 3 in patients with multifocal motor neuropathy (MMN), amyotrophic lateral sclerosis (ALS), and healthy controls (HC) (represented as 95% quantile).

| **NT (number of tracts)** | **MMN** | **ALS** | **HC** |
| --- | --- | --- | --- |
| **Entire nerve** | 320***** | 326***** | **779*** |
| **Segment 1** | 9283 | 7205 | 7172 |
| **Segment 2** | 2920 | 1713 | 4561 |
| **Segment 3** | 1075 | 1138 | 1618 |

^*^Significant difference in NT between entire nerve and segment 1, 2 and 3.
